# Supplementary material for: Expression and purification of human neutrophil proteinase 3 from insect cells and characterization of ligand binding
Source: PLoS One. 2024 Jun 25;19(6):e0294827. doi: 10.1371/journal.pone.0294827 (PMC11198849; doi:10.1371/journal.pone.0294827)
Supplement: S1 Raw images — (PDF) [file pone.0294827.s002.pdf]

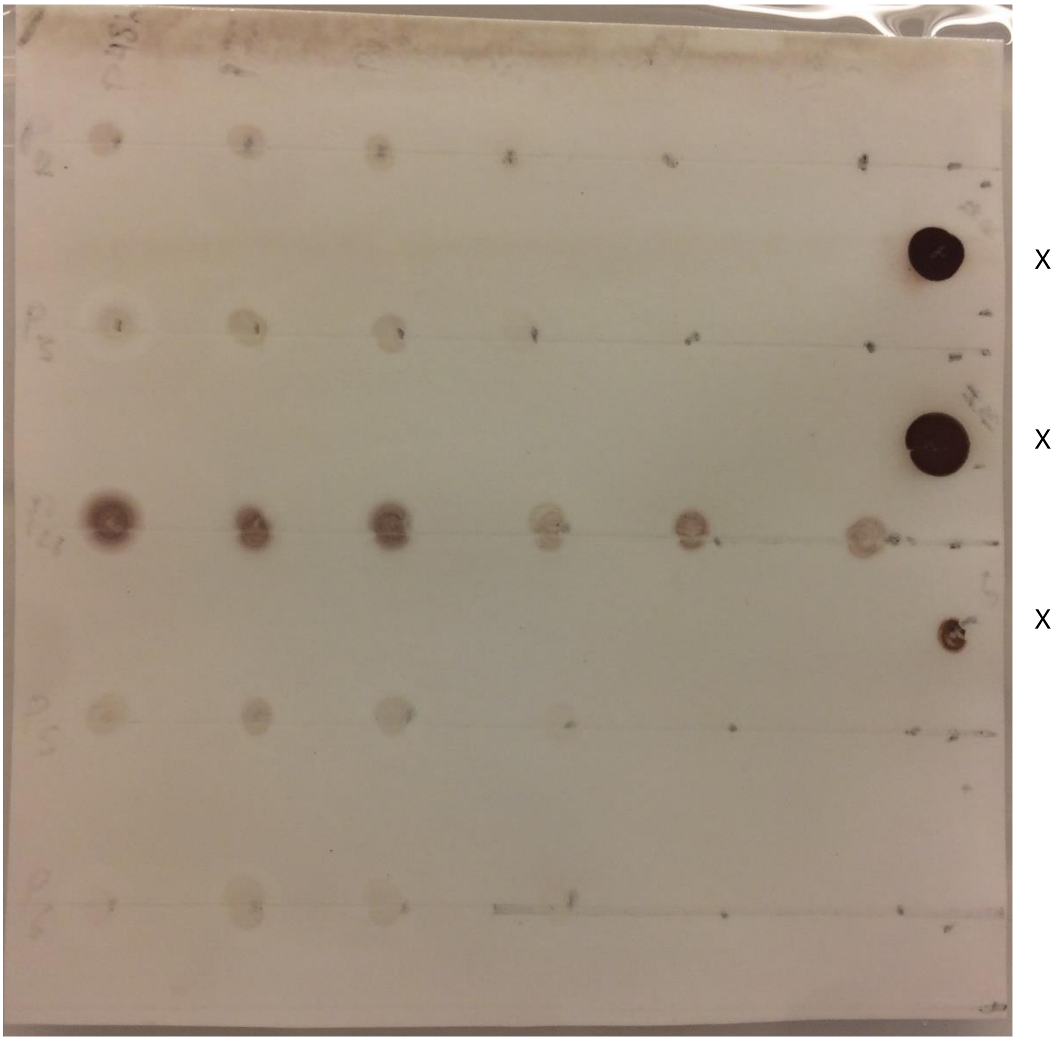

Original blot used to generate Fig 2a- Dot blot analysis of rPR3 expression. The blot a was treated with an anti-His antibody. The photo was taken with an Iphone camera. The two large dark brown dots on the right which are not shown in Fig 2a are positive controls (panthothenate kinase containing a His-tag). The smaller large dark brown dots stems from Myo-A containing a Strep-tag. It is unclear why this protein was labled with the anti-His antibody. However, as none of the other samples containing a Strep-tag were labelled with this anitbody, but they were labelled with an anit-Strep-tag antibody (Fig 2b), this was not further investigated.

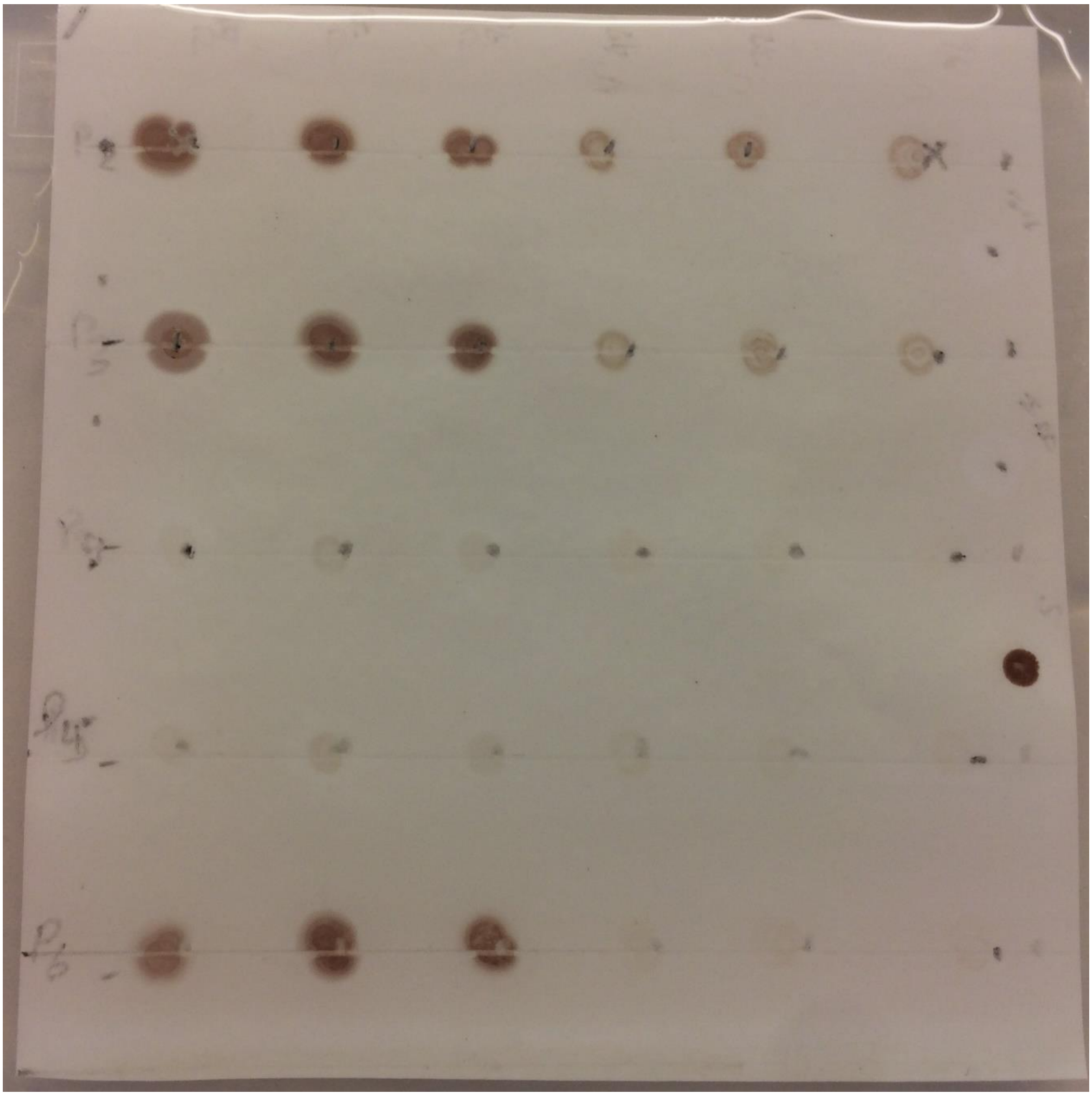

X

Original blot used to generate Fig 2b- Dot blot analysis of rPR3 expression. The blot was treated with an anti-Strep-tag antibody. The photo was taken with an Iphone camera. The dark brown dot on the right which is not shown in Fig 2b is a positive control (Myo-A containing a Strep-tag).

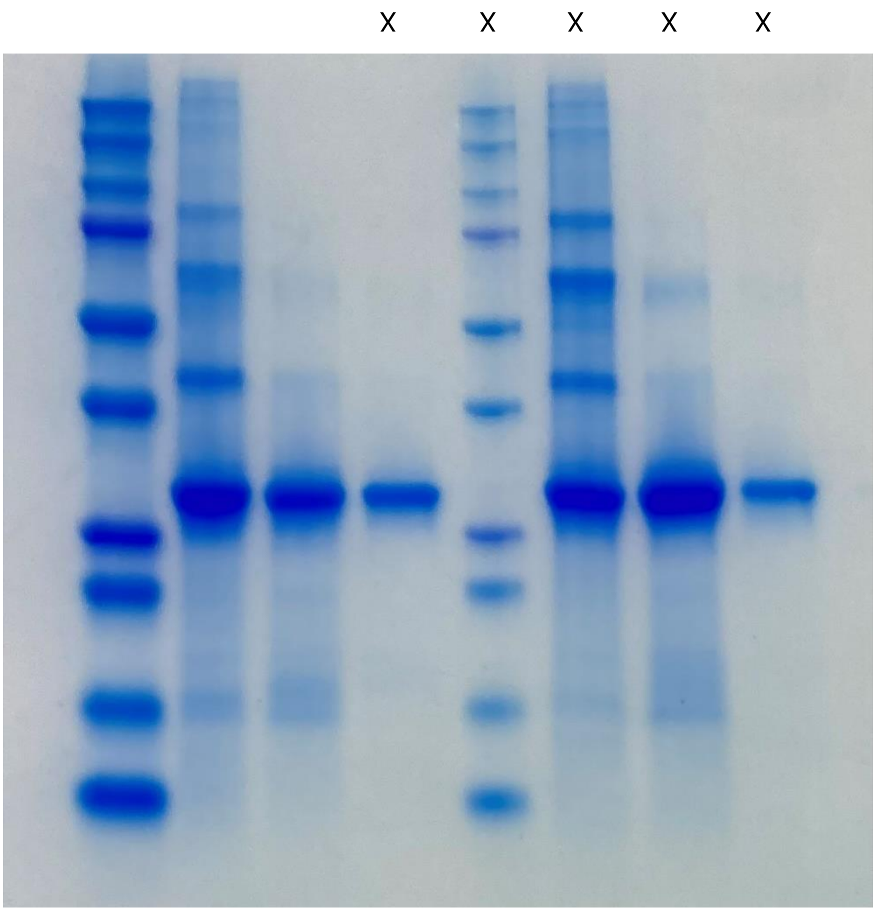

Original SDS-PAGE image used to generate Fig 3- SDS-PAGE showing the purity of rPR3 and Fig S1c- Expression and identification of rPR3. The first three lanes from the left are shown in Fig 3 and Fig S1a (Bio-Rad Precision plus protein standards (Cat# 1610374, 10-250KD, 7 μL loading), sample from Excel His-trap column purification (5μg) and SEC200 column purification (3μg), respectively). The lanes marked with "X" are repeats from the other lanes and therefore not shown in Fig 3 and Fig S1c. The gel was treated with Coomassie staining. The photo was taken with an Iphone camera.

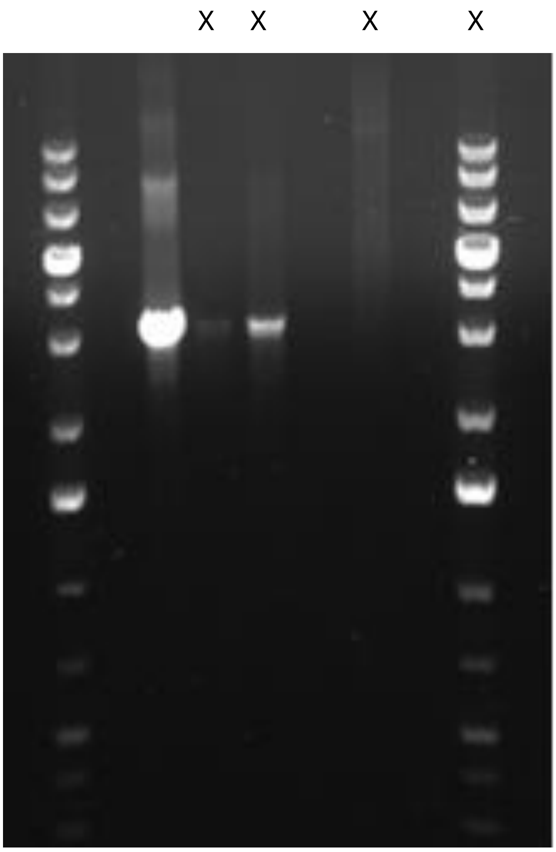

Original agarose gel image used to generate Fig S1a- Agarose gel to detect bacmid production. Only the two first lanes from the left are shown in Fig S1a**.** The first lane from left contains ThermoScientific GeneRule 1Kb plus DNA ladder and the second lane the bacmid. The image was taken with a Bio-Rad gel doc XR system.
